# The New Volume

**Published:** 1893-03

**Authors:** 


### *EDITORIAL.*

---

The office of THE JOURNAL is in room 330 Equitable Building.
Address communications relating to the Editorial Department to DR. L. B. GRANDY, Atlanta, Ga., Box 431.
Business communications should be addressed to DR. M. B. HUTCHINS, Atlanta, Ga.
All remittances should be made payable to "ATLANTA MEDICAL AND SURGICAL JOURNAL."
Articles for publication should reach this office not later than the 15th instant.
The editors are not responsible for views expressed by contributors.

---

### THE NEW VOLUME.

---

With this issue THE JOURNAL enters upon its twenty-seventh year of publication, or the tenth year of the new series. It has been our pleasure to be associated with it for the past two years, and during that time we have endeavored to prepare for our readers a high-class medical journal, the equal of any published in this country at the same price. We here present our acknowledgments to those of our friends who have been kind enough to tell us, or to write us, that our efforts have been successful. We have received many gratifying assurances on this point for which we are greatly obliged. While we ourselves believe that THE JOURNAL has improved much within the last two years, we have been, and are still, distinctly aware of many respects in which improvement is both possible and desirable. In knowledge of this fact we inaugurate this month a system which will greatly enhance the value of THE JOURNAL. The department of "*Selections and Abstracts*" will hereafter be divided into sections, and each of these latter will be in charge of gentlemen specially fitted by experience for the control of the sections which they have agreed to edit. And we may take this occasion to acknowledge our indebtedness to these gentlemen for the kind assistance which they have promised. It will be the effort of our entire staff to place THE JOURNAL on a plane superior to that of any other in the South.

We would like to see established in this section a high class medical journal which will be the medium of communication between the doctors of this section especially and the profession at large. If we shall be at all instrumental in achieving this result, we shall be much gratified. Southern doctors write too little. They lack neither the talent nor the experience, but they will not take the time to record what their experience and observation have taught them. In our efforts in behalf of THE JOURNAL we can be materially encouraged and assisted by our readers and friends. Send us your papers, speak a good word about THE JOURNAL to your brother physician who may not be a subscriber, and lastly, but very importantly, pay your own subscription if you are behind in it.

The condition of THE JOURNAL to-day is better than ever before. Our subscription list is being constantly added to, and our advertising pages were never so well patronized. We will not stop here. The feature which we now inaugurate will be an important step in advance ; and one which will secure a wider circulation and a more general and more generous appreciation.

